# Effect of receiving mobile text messages on cortisol concentrations in students at the University of the Free State

**DOI:** 10.4102/hsag.v28i0.2064

**Published:** 2023-02-23

**Authors:** Roné Vorster-De Wet, Anthonie M. Gerber, Jacques E. Raubenheimer

**Affiliations:** 1 Department of Basic Medical Sciences, Faculty of Health Sciences, University of the Free State, Bloemfontein, South Africa; 2 Department of Biostatistics, Faculty of Health Sciences, University of the Free State, Bloemfontein, South Africa; 3 Department of Biomedical Informatics and Digital Health, Faculty of Medicine and Health, The University of Sydney, Sydney, Australia

**Keywords:** mobile phone, texting, student learning, salivary cortisol, stress, anxiety, depression

## Abstract

**Background:**

Texting has become central to social life, with adverse effects on physiological functioning. Research into the impact of texting on cortisol secretion is limited.

**Aim:**

Thus study aimed to determine how receiving mobile text messages affected salivary cortisol concentrations and investigate the moderating effects of stress, anxiety and depression on cortisol secretion.

**Setting:**

Undergraduate physiology students attending physiology lectures at the Faculty of Health Sciences, University of the Free State, 2016.

**Methods:**

An experimental, crossover, quantitative design was used. Participants were involved over two consecutive days, receiving mobile text messages (intervention) on one day and acting as their own control on the other. Self-reported data on stress, anxiety, depression and subjective experience of the study, and saliva samples were collected. Text frequency and wording (neutral, positive, negative) were varied among participants.

**Results:**

Forty-eight students participated in the study. Salivary cortisol concentrations did not differ significantly between the intervention and control days. High anxiety levels were associated with increased cortisol concentrations. No associations with cortisol concentrations were documented in low to moderate anxiety, stress, depression or how participants experienced the intervention. There were no significant differences between text frequency, text emotion and change in cortisol concentrations on the intervention day.

**Conclusion:**

Receiving mobile text messages did not elicit a significant cortisol response in participants.

**Contribution:**

Findings added to the body of knowledge about the effect of texting on student learning by measuring salivary cortisol concentrations in a lecture setting, with investigation into the moderating effects of stress, anxiety, depression and participants’ subjective experience.

## Introduction

Mobile phones are an integral part of everyday life. A recent report by the Pew Research Center (Silver & Johnson 2018) indicated that 91% of adults in South Africa own a mobile phone, with its use gravitating towards social and entertainment purposes. Text messaging was one of the most common mobile activities. Mobile phones are known to be very popular among university students. Research into the use of mobile phones by South African university students revealed the patterns of use in their daily lives (North, Johnston & Ophoff 2014). United States studies on the impact of mobile phone use in class showed that, despite several benefits of mobile phone use, texting was shown to limit students’ cognitive capacities in lecture settings and be a distractor for lecturers and fellow students (Kuznekoff & Titsworth 2013). Students who text messaged throughout the lecture had significantly lower academic outcomes than the control group (Dietz & Henrich 2014). Frequent text messaging during class was found to be negatively related to academic performance (Junco 2012).

The content of text messages may elicit various psychological responses. Research showed that text perceived as hurtful was directly related to distancing behaviour and relationship satisfaction was negatively associated with perceived intent and distancing (Borae 2013). Using a positivity strategy via texting predicted satisfaction in romantic relationships and friendships (Brody & Peña 2015). No significant difference was found between men and women in recalling positive and negative words in general (Cuming 2013). Gender analysis among university students revealed that women showed greater signs of addiction to their mobile phones than men (North et al. 2014).

Although the negative impact of texting on academic performance is well established and patterns of mobile phone use by South African university students have been investigated, there is no definitive literature on the psychological impact of texting in the lecture setting.

Salivary cortisol may (or may not) indicate anxiety. Salivary biomarkers could be helpful in the quick diagnosis of stress, anxiety and depression (Chojnowska et al. 2021). Stressful stimuli activate the hypothalamus-pituitary-adrenal cortex axis (HPA) and lead to an increase in cortisol secretion (Adam & Kumari 2009). Salivary cortisol represents free unbound cortisol (5% – 10% of total cortisol) and offers a possible evaluation of free steroid hormone concentrations in the ambulant setting (Kudielka et al. 2012). Furthermore, despite several challenges, salivary cortisol measures are increasingly being incorporated into epidemiological research because of important advantages (Adam & Kumari 2009). The effect of texting on salivary cortisol concentrations during an academic lecture in the South African population is unknown.

## Aim and objectives

The study aimed to add to the body of knowledge about the effect of texting on student learning. In this context, the possible interaction between the distractibility of receiving mobile text messages and increasing stress was investigated by measuring salivary cortisol concentrations within a South African undergraduate student population in a lecture setting.

Objectives were to:

- determine the general and texting characteristics of the sample population
- investigate the moderating effect of stress, anxiety and depression on cortisol secretion while receiving text messages
- investigate whether subjective feedback on the experiment correlated with cortisol data
- investigate the moderating effect of text frequency and text emotion on cortisol secretion.

## Methods

### Study design

The study was an experimental, crossover, quantitative study over the first 6 months of 2016. The target population for this study was undergraduate students registered for the Physiology module in the Faculty of Natural and Agricultural Sciences, University of the Free State (UFS). Men and women aged 18–25 years were included in the study. Participants with adrenal gland disease or who used corticosteroid medication in the form of a cream, oil, pills or any other substance containing corticosteroids were excluded.

To address the complexity of obtaining consent from both the lecturer and students and because of the practicalities surrounding the saliva sample collection, participation was limited to students attending physiology lectures scheduled at the Faculty of Health Sciences, UFS, on two consecutive days. The lecture hall was in the same building as the laboratory for the enzyme-linked immunosorbent assay (ELISA) analysis. This simplified the collection, storage and analysis of the samples. From the students who completed the consent forms, 50 students were randomly selected to participate; however, only 48 students adhered to the study protocol and completed the study. Sample size was limited by budgetary constraints – a common factor in such studies, with Clements (2013) noting that ‘Studies using biological markers such as cortisol are often underpowered, possibly because of the expense of analysis or refusals by participants.’ Where possible, we report effect sizes to account for the small sample.

Students participated in the study over two consecutive study days at the same time slot (English lectures on Thursday and Friday from 09:10 to 10:00; Afrikaans lectures on Monday and Tuesday from 08:10 to 09:00). Participants were informed of the date of intervention but were blinded to the time of messaging and the content of the messages. Students were randomly assigned to receive the intervention on either the first or second day. On the intervention day, predetermined text was sent by the researcher to the assigned students during the lecture. The predetermined text was composed of words with a positive, negative or neutral connotation previously tested in the South African context (Cuming 2013). Positive wording was randomly assigned to 19 students, negative wording to 16 students and neutral wording to 13 students. Students received either 10, 15 or 20 text messages over 20 min. Text messages were sent to assigned students simultaneously using BulkSMS. For control, the previous or next day was texting free for the student. Each participant was provided with a dedicated mobile phone for the purpose of the study to avoid confounding factors.

### Salivary cortisol evaluation

Saliva samples were collected 10 min after commencement of the lectures (the ‘before’ sample) and again at the end of the lectures (the ‘after’ sample), at the same time of day, on both the intervention day and the control day. The after samples were collected no earlier than 20 min after the intervention. Each participant provided four saliva samples of at least 1 mL per sample. Samples were stored at −20 °C until analysis. Analyses were performed using the IBL International Cortisol Saliva ELISA kit. After the enzymatic reaction was stopped, optical density was measured within 5 min using a photometer at 450 nm wavelength. Mean measured optic density values for calibration were in range of the kit validation results. The control concentration of 0.0454 mg/dL was shown to be below the lower limit of validation (0.053 µg/dL). All samples were tested in duplicate. No outliers were observed. All standard deviation values were < 0.1, and all *R*-squared values were > 0.99.

### Questionnaires

Questionnaires were used to collect demographic data and to measure stress, anxiety, depression and participants’ subjective experience of the study. Two questionnaires were compiled from validated scales and previous literature.

The first questionnaire included information from the Hospital Anxiety and Depression Scale (HADS) to detect states of anxiety and depression (Zigmond & Snaith 1983). Thus, before the intervention and on the control day, demographic data were collected, and participants self-assessed lifestyle, stress, anxiety and depression using the HADS. Closed-ended questions to screen participants were included. These questions measured refraining from strenuous exercise, this included refraining from alcohol and non-prescription medication (24 h pre-study), caffeine (6 h pre-study), eating or drinking (other than water) (hour before and during the study) and smoking and brushing teeth (30 min pre-study).

Even though the HADS initially was designed for the hospital setting, *t* has been proved a good instrument for use in the general population (Elzinga et al. 2008). The HADS comprised subsections for anxiety (HADS-A) and depression (HADS-D). For each subsection, total scores were calculated as the sums of items. Anxiety levels were categorised as ‘normal’ if the total score was 0–7, ‘borderline’ if 8–10 and ‘abnormal’ if 11–21. In a review, Bjelland et al. (2002) report that across 15 studies, Cronbach’s *α* ranged from 0.68 to 0.93 for HADS-A and from 0.67 to 0.90 for HADS-D.

The 10-item Perceived Stress Scale (PSS-10) was added to determine to what extent situations in the student’s life are appraised as stressful (Cohen, Kamarck & Mermelstein 1983). Total score was calculated as the sum of items, ranging from 0 to 40. Higher total scores indicate greater levels of stress. In our study, total scores of less than 14 were considered low stress levels, 14–26 moderate stress levels and more than 26 high stress levels. Cohen et al. (1983) found Cronbach’s *α* values from 0.84 to 0.86 for three different samples, with test–retest reliabilities of 0.85 and 0.55 in two samples.

On the intervention day, participants completed a second questionnaire on their subjective experience of the study. On this day, five questions were put to the participants. The response to the questions was based on a five-point rating scale. Participants specified their level of agreement to a question as follows: (0) strongly disagree, (1) disagree, (2) undecided, (3) agree and (4) strongly agree.

### Statistical analysis

Data analysis was performed by the Department of Biostatistics, Faculty of Health Sciences, UFS, using SAS/STAT software (Version 9.3 of the SAS system for Windows; Copyright © 2010 SAS Institute Inc.). Study results were listed and summarised descriptively as deemed appropriate to the nature of the variables. Categorical data were presented by frequency and percentage. Paired *t*-tests were conducted to evaluate differences. A mixed model for repeated measures was used to test the intervention’s effect on the change in cortisol scores from before to after the lectures. One-way between-subjects analysis of variance (ANOVA) was conducted to compare the effects of questionnaire scores, text frequency and text emotion to salivary cortisol concentrations by category. Because of the small sample size, *h*^2^ effect size values of approximately 0.2 were viewed as small effects, approximately 0.15 as medium effects, and approximately 0.26 as large effects (Bakeman 2005). Independent *t*-tests were conducted to compare changes in salivary cortisol concentrations to the participants’ subjective experience of the study. Confounding was limited at the design level by using students of approximately the same age in a uniform testing context. The other possible confounders of sex and ethnicity were measured but were not found to be independently associated with any of the outcomes. For simplicity, all results are shown without including sex or ethnicity.

Deviations reported by the participants from the study restrictions were considered minor, and the data of these participants were included in the data analysis.

### Ethical considerations

The study was approved by the Health Sciences Research Ethics Committee, Faculty of Health Sciences at UFS [HSREC 116/2016]. Ethical clearance was also obtained from university management, faculty management, heads of department and lecturers as relevant. Informed consent was obtained from participants before any study-related procedures were conducted. National legal requirements apply to participants’ confidentiality, personal information, study findings and data archiving.

## Results and discussion

### General and texting characteristics of the study sample

Two of the 50 randomly selected participants did not adhere to the study protocol and were removed, and 48 participants completed the study (Table 1). The average age in this study was within the scope for 2-year students at South African institutions of higher education.

**TABLE 1:** Demographics of the study sample.

Variable	*N*	%	Mean age	± SD
**Gender**				
Male	22	45.8	20.5 years	± 1.34
Female	26	54.2	20.7 years	± 1.69

Nearly all participants owned a mobile phone (97.9%). This is more than the 91% reported for adults in South Africa (Silver & Johnson 2018). The 2018 Pew Research Center report found that people with a higher educational status are more likely to own a mobile phone. In Kenya, it was found that 95% of higher educated people owned a mobile phone, which supports the finding in this study (Silver & Johnson 2018). Even though not all participants owned a mobile phone, they all knew how to operate a mobile phone. Most of the participants in our study reported that they seldom or never send (87.5%) or receive (72.3%) text messages during lectures. In a study conducted among students mainly at the University of Cape Town (North et al. 2014), most respondents indicated that they did not receive calls during a lecture and found it annoying if other students made or received calls during lectures.

### Effect of texting on cortisol secretion

In this study, salivary cortisol concentrations were higher on the intervention than on the control day although not significantly. The mixed model showed that the mean salivary cortisol concentrations were significantly higher in the post-lecture than the before samples (*F* = 63.07, *p* < 0.0001) with no significant effect for the intervention and no interaction between the intervention and the pre- and post-lecture measurements. The rise in cortisol levels from before to after the lectures is in contrast to the diurnal rhythm of cortisol, which typically dictates that cortisol concentrations are high on awakening, increase to a peak 30–45 min after awakening and gradually decline throughout the day, reaching a nadir around midnight (Hall 2015). Studies similar to our study investigated the effect of momentary stressors on cortisol concentrations and found different results (Merz & Wolf 2015; Schoofs, Hartmann & Wolf 2008). Investigating the effect of oral presentations during a university course on cortisol concentrations, it was found that cortisol concentrations increased on the presentation day but declined on the control day when no oral presentation took place (Merz & Wolf 2015). Furthermore, it was found that, on average, cortisol concentrations were significantly increased on the examination versus on the control day (Schoofs et al. 2008).

The cortisol profiles observed in this study can most probably be attributed to the stressfulness of study participation, while the applied stressor (receiving text messaging) *per se* not being stressful enough to impact cortisol secretion. These findings are in line with results from a study investigating the influence of mobile phone use and availability on cortisol secretion (Hunter et al. 2018).

### Moderating effect of stress, anxiety and depression

#### The Hospital Anxiety and Depression Scale

Anxiety and depression indicators were measured on both study days using the HADS questionnaire. No significant differences between scores were observed (Table 2). This attests that participants reported scores accurately across the study days. The mean scores between the 2 days were 6.05 for the anxiety scale and 3.07 for the depression scale. This indicated that participants, on average, reported normal levels for anxiety and depression. When assessed against normative data collected for the HADS in the United Kingdom (Breeman et al. 2015) and Germany (Hinz & Brähler 2011), the participants in our study scored lower on the anxiety scale compared with the U.K. population and higher than the Germany population. Depression scores were lower than those reported in the United Kingdom and Germany. It should be observed that the studies in the United Kingdom and Germany included considerably larger samples than our study as the sole purpose of these studies was to determine normative values for the HADS. Furthermore, respondents were from various social and demographic backgrounds in contrast to a more homogeneous sample included in this study. The fact that young students who yet have to experience stressors associated with post-university life represented the majority of the participants within this study population may explain the lower depression scores compared with the normative data.

**TABLE 2:** Control/intervention paired *t*-tests.

Scale	*t*	*df*	*p*
HADS-Anxiety	1.40	47	0.1689
HADS-Depression	−0.35	47	0.7295
PSS	0.89	47	0.3776

HADS, Hospital Anxiety and Depression Scale; PSS, Perceived Stress Scale.

One-way between-subject ANOVAs (Table 3) were conducted to compare the effect of HADS-A scores on salivary cortisol concentrations by study day and anxiety level (‘normal’, ‘borderline’, ‘abnormal’). For the control day, there was no significant difference between anxiety levels and salivary cortisol concentrations (*F*_(2,45)_ = 0.77; *p* = 0.47, *h*^2^ = 0.03). For the intervention day, there was a significant difference between anxiety levels and salivary cortisol concentrations (*F*_(2,45)_ = 4.00, *p* = 0.0252, *h*^2^ = 0.15). Consequently, the Scheffé test (Scheffé 1999) indicated that participants with ‘abnormal’ anxiety levels had higher salivary cortisol concentrations than participants who reported ‘normal’ HADS-A scores. The ‘normal’ and ‘borderline’ groups did not differ significantly. Vreeburg et al. (2010) examined the association between subtypes of anxiety disorders and salivary cortisol concentrations in a large cohort study. Patients with a current anxiety disorder had higher awakening cortisol concentrations than the control group, particularly patients with panic disorder with agoraphobia and anxious patients with comorbid depressive disorder. Although there are inconsistencies in research findings, anxiety disorder has been linked to increased corticotropin release, which in turn stimulates HPA activity (Vreeburg et al. 2010). While extensive literature links major depressive disorder to HPA activity, the association between anxiety disorders and the HPA is not well established (Gilbert et al. 2017; Vreeburg et al. 2013).

**TABLE 3:** Analysis of variance for cortisol levels by levels of anxiety, depression (Hospital Anxiety and Depression Scale), stress (Perceived Stress Scale) and text frequency and emotion.

Scale	Condition	*df*	*F*	*p*	*h* ^2^
HADS-anxiety	Control (pre)	2	0.77	0.4711	0.03
HADS-anxiety	Intervention (pre)	2	4.00	0.0252	0.15
HADS-depression	Control (pre)	1	3.39	0.0719	0.07
HADS-depression	Intervention (pre)	1	2.92	0.0941	0.06
PSS	Control (pre)	2	0.01	0.9870	0.00
PSS	Intervention (pre)	2	0.11	0.8969	0.00
Text emotion	Change from pre to post intervention	2	0.09	0.9103	0.00
Text frequency	Change from pre to post intervention	2	2.45	0.0980	0.10

HADS, Hospital Anxiety and Depression Scale; PSS, Perceived Stress Scale.

One-way between-subject ANOVAs (Table 3) were conducted to compare the effect of HADS-D scores on salivary cortisol concentrations by study day and depression level. No participants reported ‘abnormal’ HADS-D scores and no significant differences were observed between HADS-D scores and cortisol concentrations. In contrast, a significant association between HADS-D scores and cortisol concentrations was found in young Greek adults living in a stressful social environment (Faresjö et al. 2013). In depression, hyperactivity of the HPA is mainly observed; however, in some patients, hypoactivity is observed, most probably as a result of HPA fatigue resulting from various depressive episodes (Vreeburg et al. 2013). Anxiety often precedes major depressive disorder (Adam et al. 2014). As these disorders share clinical features, it was proposed that there may be a common physiological basis to the disorders (Vreeburg et al. 2013).

#### Perceived stress scale

In this study, stress was measured on the intervention and control days using the PSS-10. There was no significant difference between scores reported on the respective days, indicating accurate reporting between the study days (Table 2). The mean score between the 2 days was 17.06, indicating that the participants, on average, reported moderate stress levels. Although the sample population was rather small, the reported stress levels of the intervention group were concerning (*n* = 31; 65%). Stress levels were measured in 524 social science students in the United Kingdom using the 10-item PSS; the mean reported score for the sample was 19.79 (Denovan et al. 2019). Participants in this study were less stressed than their contemporary peers attending university in the United Kingdom. The small sample in this study might explain the inconsistent findings. Another important factor to consider is the ethnic diversity of the South African population and how stress affects different ethnic groups in South Africa.

A one-way between-subjects ANOVA (Table 3) revealed no significant differences when comparing the effect of PSS-10 scores on salivary cortisol concentrations by study day and stress level. In a study among nursing students, Akbari, Gharehzad Azari and Mousavi (2017) measured the relationship between spiritual well-being and stress, anxiety and depression with cortisol concentrations. Likewise, they did not find any meaningful correlation between stress scores and cortisol concentrations (Akbari et al. 2017). In contrast, higher cortisol concentrations were found in healthy and working middle-aged women with higher perceived stress and depressiveness (Faresjö et al. 2014).

#### Participants’ subjective experience

Participants completed the subjective experience questionnaire on the intervention day and rated each question. In this study, 79% of participants agreed that text messaging caused distraction from the lecture. In an earlier study (Tossell et al. 2015), students who had never owned a smartphone were given one for an entire year. At the start of the year, 63% believed that the smartphone would play a large part in their academic achievement. By the end of the year, students had a negative perspective of mobile phone use in the academic setting, citing that they had become addicted to the mobile phone and distracted from their education. Kuznekoff and Titsworth (2013) stated that learning is a process, and when there is a resource that competes with the process of learning, it has a negative effect on learning. Furthermore, texting causes a divide in attention that distracts attention from the on-task behaviour, that is learning. It has been found that students feel relatively neutral about using a mobile phone in the classroom. This is despite recognising that texting causes a distraction in an academic environment (Berry & Westfall 2015). In another study (Braguglia 2008), 77% of business students believed that learning in the classroom was seldom or never affected by mobile phones, while 76% believed it seldom assisted learning, However, it should be noticed that this study was conducted in the spring semester of 2007 (January–May), and the first true smartphone, the iPhone, was released in the United States in June 2007. This is apparent because while 76% of Braguglia’s respondents had internet-enabled phones, the most frequent phone uses were voice calls (66%) and texting (30%), and only 3.6% reported internet activity as their most frequent phone use. In this study, 52% of participants reported that they anxiously waited for the text messages, while 71% wished the text messages would stop. Only 10% of participants found the experience stressful, supporting the salivary cortisol responses seen in this study. Independent *t*-tests were conducted by questionnaire item to compare the change in salivary cortisol concentrations with responses of agreement and disagreement, respectively (Table 4). These comparisons yielded no significant differences. For statistical analysis purposes, agreement was defined by ‘strongly agree’ and ‘agree’ responses to the questions posed in the participants’ subjective experience questionnaire, while disagreement was defined by ‘strongly disagree’, ‘disagree’ and ‘undecided’ responses.

**TABLE 4:** *T*-tests for cortisol level changes by positive or negative experiences (*N* = 47).

Experience	*t*	*df*	*p*
Text distracts from lecture	1.54	46	0.1304
Anxiously wait for text	0.79	46	0.4347
Wish texts would stop	0.56	46	0.5792
Find experience stressful	0.90	46	0.3722
Feel different about texting	1.09	46	0.2820

#### Text frequency and text emotion

Frequency, content and wording of text messages are important in mobile health intervention studies (Tomlinson et al. 2013). This study explored how text frequency would affect cortisol secretion. Results (Table 2) showed that text frequency did not significantly affect the change in salivary cortisol concentrations on the intervention day. Participants received either 10, 15 or 20 text messages during the intervention.

Exploring how words with a positive, negative or neutral emotional connotation would affect salivary cortisol concentrations, this study revealed that text message content did not significantly impact cortisol secretion on the intervention day. Literature regarding the content of text messages and cortisol concentrations is limited. The impact of text message content was investigated in adolescents. It was shown that text messages with affective attitudes regarding physical activity increased energy expenditure during a 2-week period compared with the control group who received neutral text messages (Sirriyeh, Lawton & Ward 2010). Research revealed that texting is an accepted form of support in adolescent obesity interventions, with respondents reporting that positive and encouraging text messages are supportive, whereas text messages that mention unhealthy foods or behaviours acted as a trigger to consume the unhealthy food and engage in the unhealthy behaviours (Woolford et al. 2011).

### Study limitations

This study was conducted at a single university using only participants enrolled for physiology modules. Selection bias should be considered when interpreting the results of this study, as all participants were physiology students.

## Conclusion

Texting has become central to social life, especially among young people. This study aimed to narrow down the physiological effect of receiving mobile text messages in a lecture setting by exploring multi-faceted variables. The primary goal was to determine how receiving mobile text messages affected salivary cortisol concentrations. In addition, the moderating effects of stress, anxiety and depression on cortisol secretion were investigated. Participants’ subjective experience of the study was compared with cortisol response. Our study also incorporated text frequency and text emotion into the study design.

Regarding the general and texting characteristics of the study population, men and women 18–25 years of age were included in the study. Nearly all participants owned a mobile phone, and all participants knew how to operate a mobile phone. Most of the participants in this study reported that they seldom or never send or receive text messages during lectures.

In this study, it was observed that the salivary cortisol concentrations were higher on the intervention than the control day although not significantly. Mean salivary cortisol concentrations were significantly higher after the lecture than before on both the intervention and the control day, indicating that the lectures may have caused stress among students.

Stress and depression did not have a moderating effect on cortisol secretion while receiving mobile text messages. Although our study indicated that anxiety levels had a moderating effect on cortisol secretion, it was the participants who reported abnormally high anxiety scores who also had higher salivary cortisol levels. Participants provided subjective feedback on their experience of the study by answering relevant questions. Despite the majority of participants agreeing that text messaging caused hindrance during lectures, reported answers compared with cortisol responses yielded no significant differences. Text frequency and negative or neutral wording had no moderating effect on cortisol secretion.

In summary, it can be concluded that receiving mobile text messages during a lecture did not significantly affect cortisol secretion, and the mediating factors that were analysed in this study did not have significant mediating effects on cortisol secretion. Nonetheless, this was the first study to measure the effect of receiving mobile text messages on salivary cortisol concentrations during a lecture. As far as could be ascertained, this study was the first to vary text frequency in a lecture setting and compare frequency to salivary cortisol concentrations.

Texting forms part of the daily lives of millions of people, therefore, it is important to continue research on the psychological and physiological effects of texting. Currently, research is limited.
